# Cellulose elementary fibril orientation in the spruce S_1-2_ transition layer

**DOI:** 10.1038/s41598-019-40303-4

**Published:** 2019-03-07

**Authors:** Mehedi Reza, Carlo Bertinetto, Kavindra Kumar Kesari, Peter Engelhardt, Janne Ruokolainen, Tapani Vuorinen

**Affiliations:** 1 Department of Applied Physics, Aalto University, P.O. Box 11100, FI-00076 Espoo, Finland; 2 Department of Bioproducts and Biosystems, Aalto University, P.O. Box 16300, FI-00076 Espoo, Finland

## Abstract

The tight organization of major wood cell wall polymers limits the swellability, solubility and reactivity of cellulose fibers during the production of regenerated textile fibers, nanocellulose, bioethanol, and many other value-added products. However, the ultrastructural assembly of cellulose elementary fibrils (EF) and matrix materials in one of the outer layers, *i.e*. S_1-2_ transition layer of wood cell wall, is far from being understood. Here, single-axis electron tomography on ultrathin spruce sections was applied to observe the three-dimensional (3D) structure of the S_1-2_ layer. The nanoscale geometries of the EFs were further quantitatively modeled through mathematical fitting of the tomographic subvolumes by suitable parametric space curves. The results showed that crisscross, bundled and parallel EF organizations are all present in this layer; the former two exhibit a denser structure. Several quantitative measures such as distances and angles were obtained for the analyzed structures. The result obtained in this study suggests that the S_1-2_ transition layer differs in structure than the principal cell wall layers. The structural differences and its possible role in wood cell wall have been discussed. These results will enhance our understanding of the swellability, accessibility and solubility of woody biomass for its conversion into the aforementioned value-added products.

## Introduction

Cellulose elementary fibrils (EFs)^1^, also known as microfibrils^2^, are embedded in a matrix of hemicelluloses and lignin may form the skeleton of wood cell walls^3–5^. Wood cellulose is composed of long microfibrils (each a few nanometers in thickness)^6^, run through a hydrated matrix of glucomannans (the dominant hemicellulose in softwood tracheids)^7^, and other polymers^8–11^. The structural organization of EFs is considered to be the prime factor that regulates the mechanical performance of solid wood on micro to macro levels and the conversion of lignocellulose fibers into various products. These value-added products could be bio-fuels, fine chemicals, and rich energy sources for microbial fermentation and enzyme production^12–15^. In the preparation of material products, fibers are often treated with chemicals and/or enzymes in order to open up the fibrillar network of the cell wall during a subsequent mechanical disintegration. However, the EF structures (EF aggregates, for example)^16^ that exist in wood and pulp reduce the accessibility of cellulose and hamper the action of enzymes and chemicals. The understanding of structural mechanics of EFs by microscopy techniques in combination with mathematical modeling would help us to explore the aforementioned value-added products. Specifically, the complex assembly of the cell wall limits its swellability, solubility and reactivity in the manufacture of micro/nanofibrillar cellulose. Also, dissolution of cellulose as a polymer for regeneration of textile fibers, and enzymatic hydrolysis of cellulose and hemicelluloses into sugars for their subsequent fermentation, for example, to ethanol to replace fossil fuels in the future^17,18^. Many studies have already suggested that the fiber ultrastructure along with solvent quality provides the most impact on the fiber swelling and dissolution process^19,20^, although, so far, the ultrastructure of the transition layers has not been studied due to their small width. However, it is well reported that fibers with secondary cell wall contains crystalline cellulose^21,22^, though its diameter (small width) and crystalline material are key inputs for the better understanding of cellulose microfibrils in respect to mechanical accomplishment^23–25^ and future outcomes.

**Figure 1:**
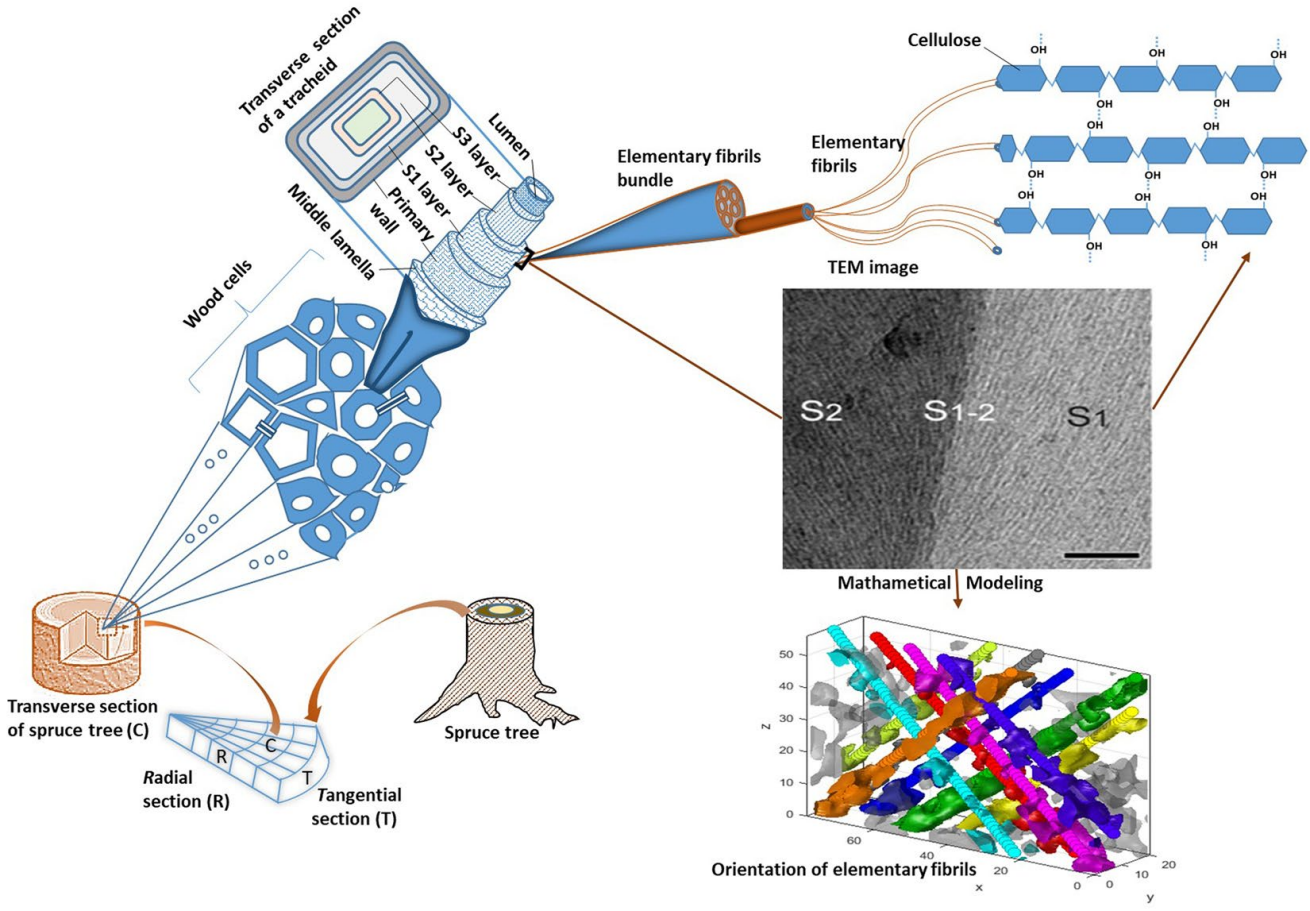
An overview of sectioning of the wood, selection of area, tomography and modeling of fibril orientation. Wood cell wall depicts layering and transmission electron micrograph shows the cellulose fibril orientation in the S_1_ and S_2_ layer; scale bar 100 nm. Wood fiber comprises thousands of elementary fibrils (EF) and their bundles. Each EF consists of several parallel cellulose molecular chains.

Current studies mainly concentrated on the major layers of the secondary wall, and to our knowledge no study has been conducted exclusively on the thin S_1-2_ layer (*i.e*. transition between the S_1_ and S_2_ layer, see Fig. 1) to understand the ultrastructural assembly of the cell wall materials. The lack of knowledge in the transition layer ultrastructure limits the understanding of its role in various applications of the cellulose fibers. Furthermore, detailed knowledge of the wood cell wall ultrastructure is imperative for mimicking the properties of wood into new synthetic materials^19^. Therefore, S_1-2_ layer ultrastructure would provide us deeper insights of the behavior of cellulose fibers in numerous applications of wood. Attempts at observing the EF orientation in the transition layers of the secondary wall were taken by using two-dimensional (2D) imaging techniques (scanning electron microscopy (SEM), for example), but this layer could not be traced due to the gradual change of EF angle from S_1_ to S_2_ layer^26^. The micrograph of spruce transverse section presented in Fig. 1, shows the orientation of EFs in the S_1_ and S_2_ layers, which is totally impossible to observe for S_1-2_ transition layer.

## Results and Discussion

**Figure 2:**
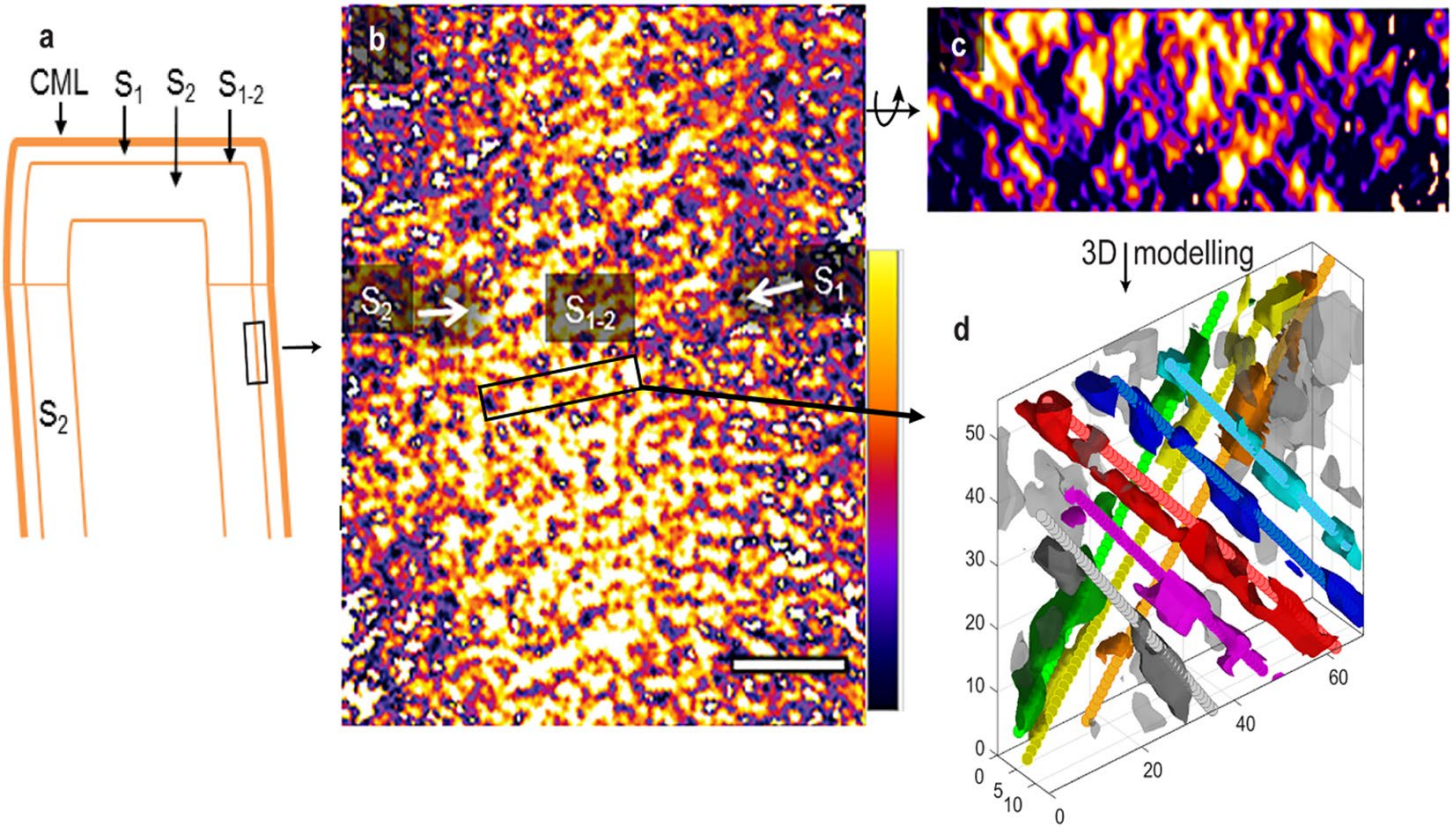
Tomography of S_1-2_ transition layer and fitting with parametric space curves. (**a**) Schematic diagram of wood cell wall; (**b**,**c**) tomographic slices of the S_1–2_ longitudinal and transverse sections, respectively, show a dense S_1-2_ layer; scale bar is 50 nm; (**d**) the space curves fit from the indicated subvolume reveal the crossed-fibrillar structure; plot units are nm. Tilt series was acquired on radial longitudinal section. The tomographic density scale bar shows low density (black color) to high density (yellow color).

**Figure 3:**
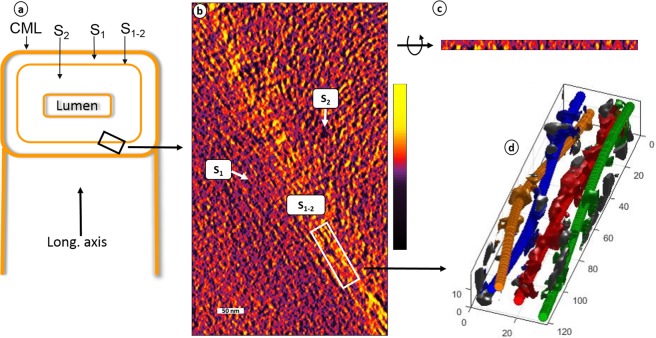
Tomography of S_1-2_ transition layer. (**a**) Schematic diagram of wood cell wall depicts transverse and longitudinal sections; (**b**,**c**) tomographic slices of the S_1-2_ transverse and longitudinal sections, respectively, show a parallel fibril orientation in the S_1-2_ layer; scale bar is 50 nm; (**d**) the resultant fitted space curves also show a parallel structure, and some fibrils appear bundled together; plot units are nm. Tilt series was acquired on transverse section. The tomographic density scale bar shows low density (black color) to high density (yellow color).

**Figure 4:**
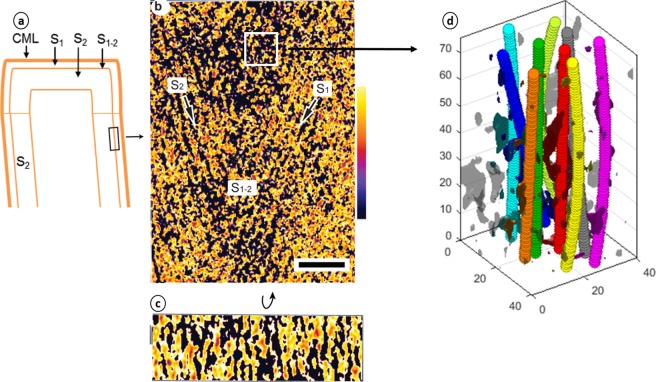
Tomography of S_1-2_ transition layer. (**a**) Schematic diagram of wood cell wall; (**b**,**c**) tomographic slices of the S_1-2_ longitudinal and transverse sections, respectively, show a parallel fibril orientation in the S_1-2_ layer (fibrils are aligned to transverse section); scale bar is 50 nm; (**d**) the resultant fitted space curves are in accordance with the parallel fibril structure; plot units are nm. Tilt series was acquired on radial longitudinal section. The tomographic density scale bar shows low density (black color) to high density (yellow color).

Cryo-transmission electron microscopy (cryo-TEM) tomography provides three-dimensional (3D) structure of S_1-2_ transition layer. 3D tomograms of this transition layer are presented in Figs 2–4. The S_1–2_ transition layer can be spotted by a drastic change of EF angle in the transverse and longitudinal sections, as shown in the electron micrographs^27,28^. These tomograms showed the existence of both crossed (Fig. 2) and parallel (Figs 3 and 4) fibril orientations in the transition layer. The crossed-fibrillar structure, which can be viewed more clearly by the fitted space curves (Fig. 2d), might originate from opposite helical orientations of EFs in the S_1_ and S_2_ layers, which cross in the transition layer forming a denser structure (Fig. 2b,c). Furthermore, the presence of a crossed-fibrillar structure is immediate (likely), when an out-of-plane orientation is present in both S_1_ and S_2_ layers with opposite helices (Supplementary Fig. S4).

Our findings suggest that an abrupt change of helices takes place in the transition layer with a crossed-fibrillar structure followed by a gradual change of EF angle in the succeeding layer. For example, EF angle in the S_2_ layer gradually changes from a high value in the outer-S_2_ to an almost axial in the inner-S_2_^26^. An almost similar EF angle of S_1_ layer with opposite helix (Z) was observed in the inner side of the S_1-2_ layer that could be belonging to the outer-S_2_ layer. It was reported that the helical organization in the secondary wall gradually changes from S-helix in the S_1_ layer to Z-helix in the S_2_ layer in the developing tracheids^26^. The gradual change of EF orientation perhaps takes place in the matured transition layers when a parallel EF orientation exists, as in Fig. 3. The EF orientation in the secondary wall region can be quite variable within individual tracheids as investigated by Donaldson and Xu^29^. An intermediate structure between the layers in secondary wall was reported in a previous study^30^. Brändström and colleagues^27^ considered the S_1-2_ transition layer as a part of the S_2_ layer. However, this study is an agreement to our previous observations^28^ that the transition layers are independent because of their unique structure.

As already mentioned, the other tomograms shown in this paper present a parallel fibril structure. A higher density can be observed in the transition layer of Figs 2 and 3, but it is not clearly evident in the one in Fig. 4. In Fig. 4c, a slight angle is visible between the EFs and the tangential plane. The transition layers usually appear brighter in the transmission electron micrographs than the principal cell wall layers^31^, indicating less lignin content in the transition layer than the other layers as lignin gets stained by potassium permanganate (KMnO_4_) and gives a dark contrast^31^. The denser structure of transition layers could be a result of EF aggregation as reported in S_2_ layer in several occasions^32^. The low concentration of lignin in the transition layer, as suggested by the electron micrographs, may trigger the cellulose fibril aggregations guided by hemicelluloses as claimed in studies on pulp^33,34^. Furthermore, hemicelluloses remain unstained by KMnO_4_ giving almost similar electron density as cellulose in the electron micrographs^31^. The tight association of EFs and their aggregation in the transition layer perhaps act as a gluing layer holding the principal cell wall layers with opposite helices to make wood strong as a material. Nevertheless, lignin is considered as the gluing agent in the intact cell wall, gluing the cells together. Several studies demonstrated that the transition between S_1_ and S_2_ layers form a weak zone, where the defibration predominantly takes place in mechanically stressed wood^35^. Investigation of the native wood cell wall presented in this study suggests a unique structure in the S_1-2_ layer that likely contributes to the structural weakness between the S_1_ and S_2_ layers. Thus, the results obtained in this study suggests several possibilities for the production of value-added products by measuring tomographical and mathematical modeling parameters to explore the future outcomes.

Fitting of the cellulose elementary fibrils (EF) in the tomographic volumes by parametric space curves shows the EF orientation in the S_1-2_ layer. The tomographic slices obtained from the S_1-2_ layer clearly show the fibrillar structure of the wood cell wall, but the modeling of such structure with manual segmentation methods^36^ seems to be challenging because of the tight association of EFs and matrix materials. To address this challenge, we fitted the tomographic density with a geometric model for the individual EFs, using an algorithm modified from the one devised by Ciesielski *et al*.^37^. In such model, EFs are approximated by parametric space curves consisting of a helicoidal term and a polynomial one (which accounts for deviations from a straight helix); the computational procedure is explained in detail in the supporting information. This method is capable of extracting the unique nanoscale geometry of individual EFs in the tomograms, with the practical effect of a resolution enhancement and providing a quantitative structural description.

For the S_1-2_ layer in Fig. 2, this model indeed showed the presence of crossed-fibrillar structure in the transition layer (Fig. 2d). The nearest neighbor distance was also calculated for the fitted curves, obtaining a mean value of ~11 nanometer (nm) among parallel EFs and much smaller (not reported, but approximately contact distance) for crossing EFs. This observation supports the idea that the dense structure in the S_1-2_ transition layer originates from the crossed-fibrillar structure. The orientation appeared rather uniform in the considered subvolumes, with nearly all fitted curves perpendicular to the longitudinal axis and showing an angle of 45° (in either direction) with the tangential plane.

For the tomogram in Fig. 3, the curve fitting revealed the presence of bundles (Fig. 3d), which might explain the higher density in this type of transition layers. In this sample, the observed bundles contain only two or three EFs, and involve around 40% of all fibrils in the transition layer. In previous studies, EF bundles have been observed in both the S_1_^38^ and S_2_ layers^34,39,40^, therefore in consideration to previous studies, it is quite obvious to find them in the transition layer of present study. The average nearest neighbor distance was ~10 nm. The fitted curves were approximately aligned to the tangential plane in the considered subvolumes, which all concerned merely the thin S_1-2_ transition layer. It will be of great interest to measure how such angle gradually changes approaching this layer, a study that will require further elaboration of the fitting algorithm. The angle between the EFs and the longitudinal axis could not be properly evaluated because of very thin sample used (less than 20 nm thick).

Bundles are not clearly observed in the tomogram of Fig. 4, although they are very difficult to detect on such a thin volume along the tangential plane. The average nearest neighbor distance was also ~10 nm; a slight decrease (~9.7 nm) is observed in the middle of the transition layer, though it may not be statistically very significant. The mathematical modeling (Fig. 4d) does not reveal a particular order apart from the parallel EF arrangement, but showed a median angle of about ±6° with the tangential plane, confirming the small angle, that was noticed by visual inspection in Fig. 4d. The angle with the longitudinal axis is perpendicular on an average, but small oscillations in either direction were observed, with a standard deviation of ~10°. The Supporting information shows plots of the spatial distribution of characteristics such as interfibrillar distances and angles throughout this tomogram. Additionally, Supplementary Fig. S1 shows average angles (degrees) of fitted EFs with the tangential plane in each square to visualize location in Fig. 4b. Supplementary Fig. S2 shows average angles (degrees) of fitted EFs with the transverse plane in each square and an overlapping of colormap visualized a location in Fig. 4b. Supplementary Fig. S3 shows Nearest neighbor distances (nm) for each analyzed subvolume, which on the *xy* plane are squares of length 40.5 and overlapping of colormap visualize a location in Fig. 4b. All about Supplementary Figs S1–S3 were described in supporting information.

## Conclusions

Transmission electron tomography combined with mathematical modeling of nanoscale geometry of cellulose elementary fibrils (EF) showed the detailed structure and orientation of EFs in the S_1-2_ transition layer. Different EF arrangements, particularly criss-crossed, bundled and parallel, were observed in the analyzed samples. The mathematical modeling also allowed for obtaining quantitative information such as nearest-neighbor EF distances, percentage of EFs forming bundles, and angles with respect to the major directions.

Wood cell wall consists of different layers like an interplay of lamellae. Having a tight arrangements of cellulose EFs, transition layers may act as a gluing layer for principal cell wall layers by forming either physical intertwingling of EFs or chemical bondings or both. Moreover, the tight association of EFs makes cellulose abundant in this particular layer, meaning that transition layers may have different cell wall materials content than the neighboring layers. All these observations on the EF structure may provide a better understanding of the reactivity of cellulosic fibers in biochemical, chemical and mechanical treatments. Further studies on wood cell wall will be necessary to get a deeper understanding of structural variation in the transition layer and its obvious role in the intact cell wall.

## Materials and Methods

### Sample preparation

In order to extract high-resolution information on the tracheid wall, a disk of Norway spruce wood was collected from breast height (~1.3 m) of a ca. 40 years old tree originating from Ruotsinkylä in Southern Finland. Cubes (3 × 5 × 10 mm^3^) of latewood were prepared without embedding in resin, before sectioning. Ultrathin sections of ~100 or 150 nm were cut from transverse and radial longitudinal wood surfaces at cryogenic temperature (−40 °C) with a diamond knife on a Leica EM FC7 ultramicrotome. A fuller description of sectioning can be found in Reza *et al*.^28^ Grids with sections were post-stained for 30 min with 1% aqueous KMnO_4_ to selectively stain for lignin followed by drying at room temperature for 2–3 hours.

### Acquiring tilt series

Nine sets of single-axis tilt series of transverse and radial longitudinal sections were acquired from −63° to +63° at 3° angular increment using SerialEM^41^ software at a pixel size of ∼0.45 nm (unbinned) or ~0.9 nm (binned 2x). Micrographs were recorded with a Gatan Ultrascan 4000 CCD camera on a cryo-TEM (Jeol JEM-3200FSC) at an accelerating voltage of 300 kV. The images were taken in bright-field mode and using zero loss energy filtering (Omega type) with a slit width of 20 eV (electron Volt). Low-dose mode of the acquisition software was used during the data collection. Specimen temperature was maintained at −187 °C during imaging.

### Tomogram assembly and visualization

Tilt series were aligned by tracking 25–35 gold markers (~15 nm) with IMOD software package^36^. Tomograms were reconstructed from the tilt series using the Simultaneous Iterative Reconstruction Technique (SIRT) within IMOD and with 10 iterations. Finally, tomographic volumes were visualized with volume viewer plugin of ImageJ^42^. Gaussian filtering within UCSF-Chimera was applied to reduce the noise to some extent^43^. In order to avoid the effect of sectioning on wood structure^44^, tomographic slices were captured from the middle part of the tomograms.

### Computational modeling

Tomographic volumes were imported and displayed in MATLAB R2017a (The Mathworks, United States of America (USA), using functions adapted from the particle estimation for electron tomography (PEET) software package^37^. Where necessary, they were rotated to approximately align the EFs with one of the axes. From each tomogram, several subvolumes were selected to perform curve fitting: precisely 13, 44 and 261 subvolumes for the tomograms in Figs 2–4, respectively, and 91 for another tomogram presenting criss-crossed fibrillar structure (not shown). Many of these subvolumes were overlapping to verify the consistency of results. The code for the fitting algorithm was acquired from Dr. Ciesielski (University of Colorado, USA) and specifically modified for this work. The description of the fitting algorithm is fully explained in the supporting information. The minimization of the cost function was performed initially using a ‘Particle Swarm Optimizer’^45^ and then refined by a simplex method with the MATLAB function ‘fminsearch’.

## Supplementary information

Supplementary Information
