# Supplementary material for: Cellulose elementary fibril orientation in the spruce S1-2 transition layer
Source: Sci Rep. 2019 Mar 7;9:3869. doi: 10.1038/s41598-019-40303-4 (PMC6405864; doi:10.1038/s41598-019-40303-4)
Supplement: Supplementary file 1 — Supplementary Information [file 41598_2019_40303_MOESM1_ESM.pdf]

Supporting Information for

Cellulose elementary fibril orientation in the spruce S_{1-2} transition layer

Mehedi Reza[†], **Carlo Bertinetto**[§], **Kavindra Kumar Kesari**[†§]*, **Peter Engelhardt**[†], **Janne Ruokolainen**[†]*, **Tapani Vuorinen**[§]*

[†] Department of Applied Physics, Aalto University, P.O. Box 11100, FI-00076 Aalto, Finland

[§] *Department of Forest Products Technology, Aalto University, P.O. Box 16300, FI-00076 Aalto, Finland*

*Corresponding Author's emails:

kavindra.kesari@aalto.fi; janne.ruokolainen@aalto.fi; tapani.vuorinen@aalto.fi

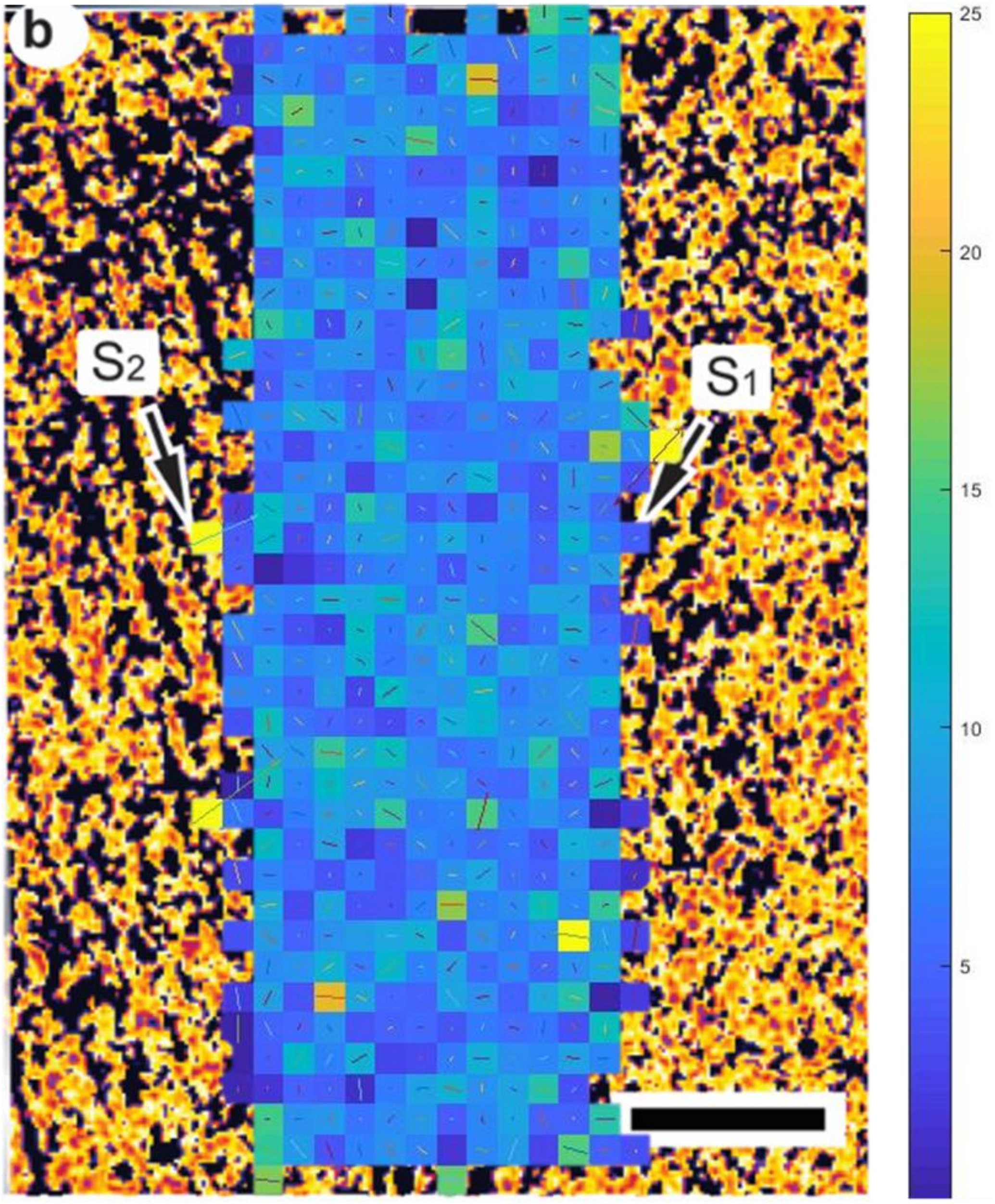

Supplementary Figure S1. Average angles (degrees) of fitted EFs with the tangential plane in each square. The colormap is overlapped on Figure 4b to visualize location. Each square has a length of 10.8 nm. The colorbar refers to the colormap in the middle of the figure (S_{12} transition layer).

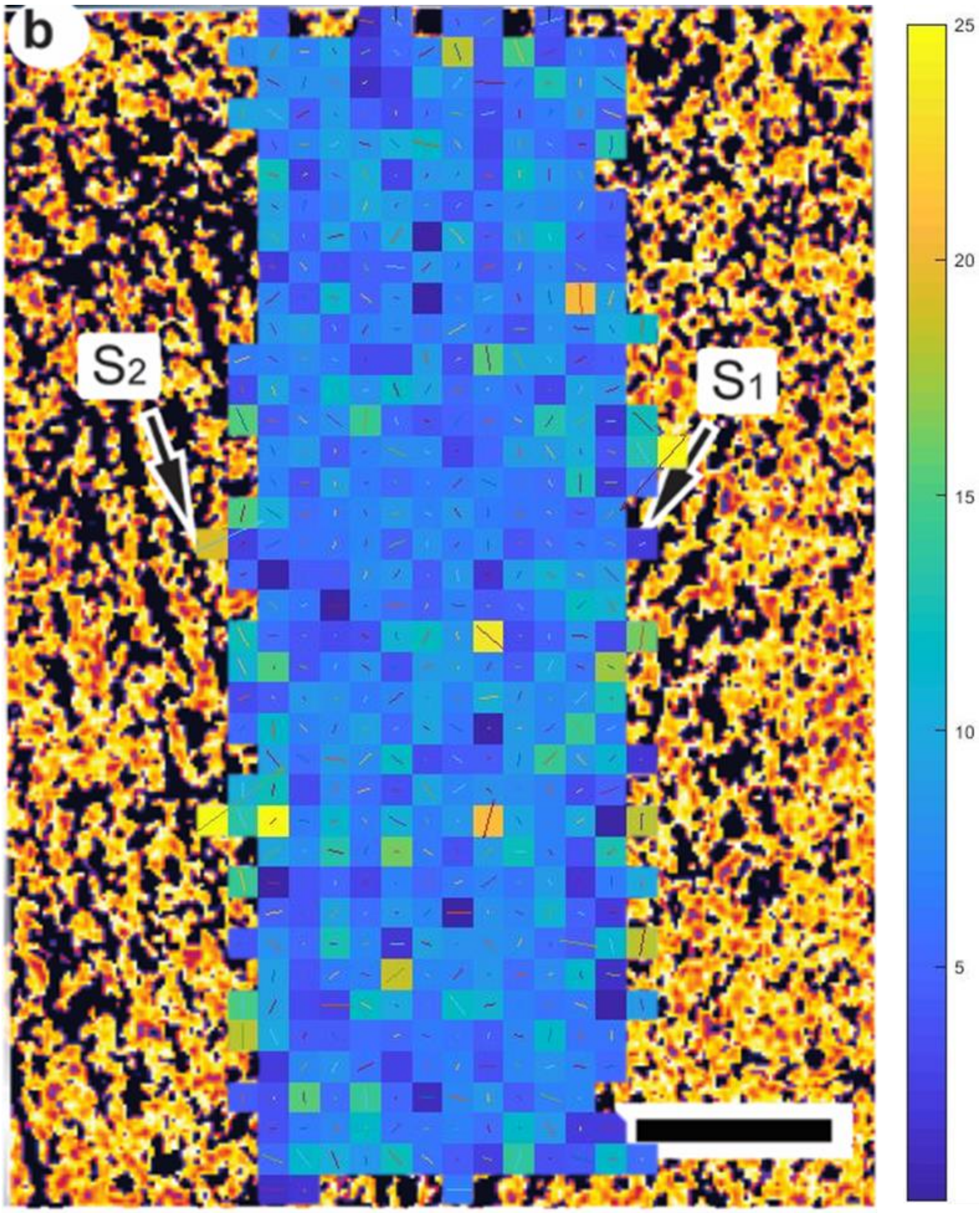

Supplementary Figure S2. Average angles (degrees) of fitted EFs with the transverse plane in each square. The angles with the longitudinal axis are complementary (sum to 90°) to the ones shown here. The colormap is overlapped on Figure 4b to visualize location. Each square has a length of 10.8 nm. The colorbar refers to the colormap in the middle of the figure (S_{12} transition layer).

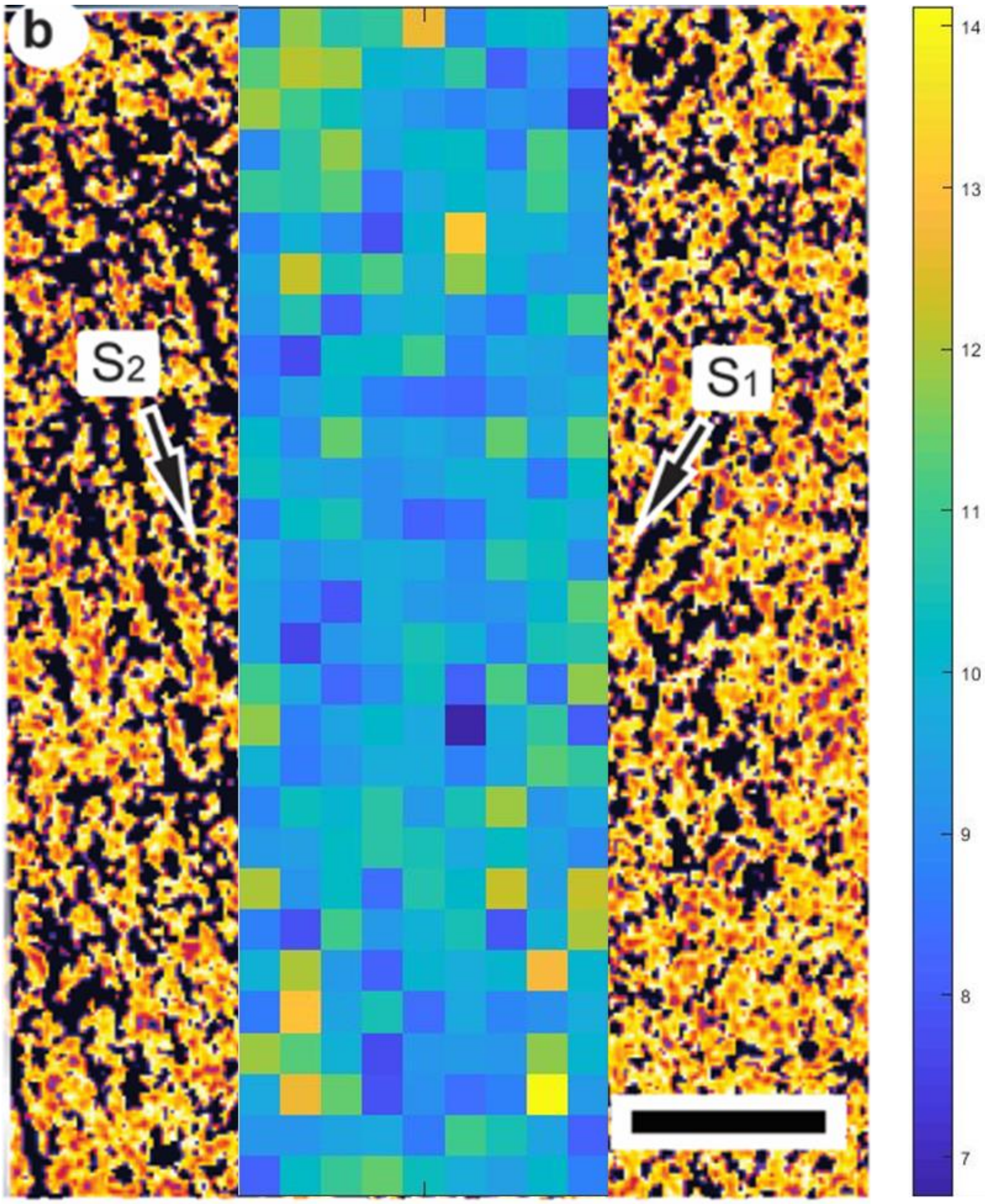

Supplementary Figure S3: Nearest neighbor distances (nm) for each analyzed subvolume, which on the *xy* plane are squares of length 40.5 nm and whose centers are located on the squares of the color map. The colormap is overlapped on Figure 4b to visualize location. The colorbar refers to the colormap in the middle of the figure (S_{12} transition layer).

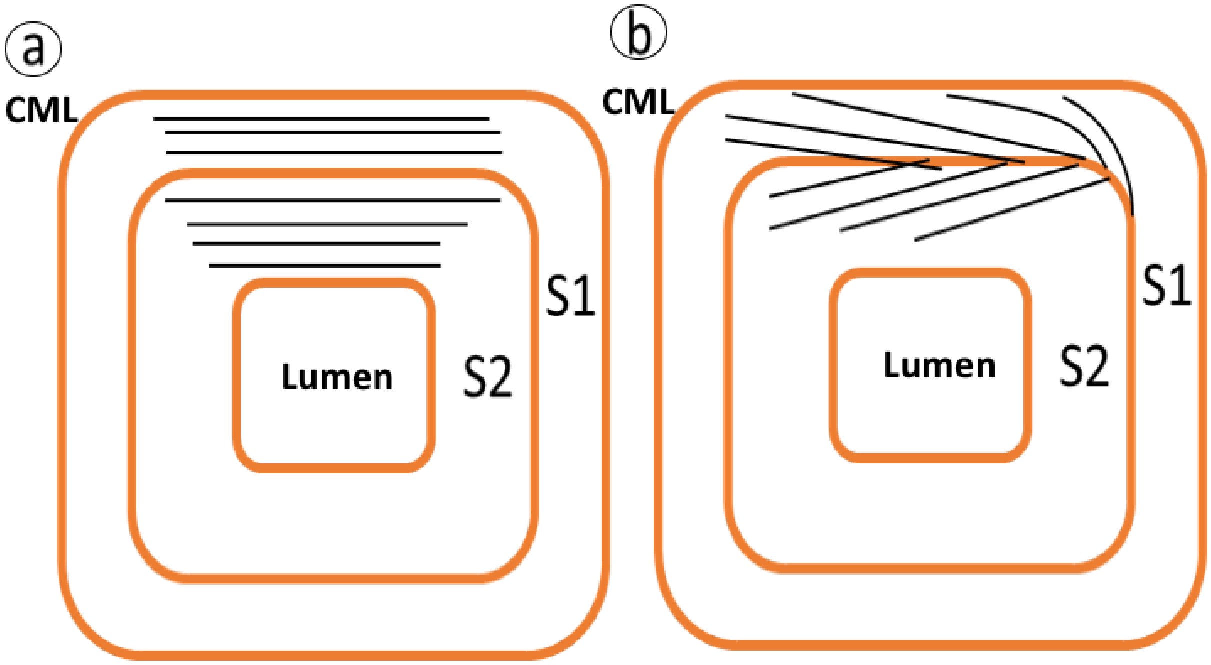

Supplementary Figure S4: Crossed-fibrillar structure: if the microfibril orientation in the S_1 and S_2 layers follows the concentric plane of wood cell wall, there is less or no possibility of crossed-fibrillar structure in the $S_{1\text{-}2}$ transition layer as shown in fig. S4a. The sketch in fig. S4b shows that a crossed-fibrillar structure is likely when microfibrils in the neighboring principal layers, with opposite helices, protrude from the cell wall plane towards the lumen.

Description of the fitting algorithm

The algorithm used in this work to fit the geometry of the EFs is an adaptation of the one presented by Ciesielski *et al.* (in ref. 37 of the main text). The selected tomographic subvolume is initially normalized and rotated so that one of the axes (here chosen to be *z*) is roughly aligned to the filamentous structural patterns that can be distinguished by visual inspection. The tomographic density is then fitted by parametric space curves of the form:

$$\mathbf{h}(t) \equiv \begin{Bmatrix} x = c_1 \sin(\omega t + \varphi) + P_x(t) \\ y = c_2 \cos(\omega t + \varphi) + P_y(t) \\ z = t \end{Bmatrix} \qquad (1)$$

Where **h**(t) is an *xyz* triple that defines the point of a space curve **h** at the parameter value of *t*. The trigonometric operators allow the space curve to adopt a helical geometry with *x* and *y* amplitudes

(c_1, c_2), angular frequency (ω), and phase shift (φ). The terms $P_x(t)$ and $P_y(t)$ are polynomials, which confer the curve flexibility to deviate from a straight helix. The algorithm places space curves in the regions of the subvolume with the highest relative density, by varying the parameters of equation (1) while optimizing an appropriate cost function.

The mass enclosed within a chosen distance L on the x and y dimensions from the curve $\mathbf{h}(t)$ is given by:

$$M_{\mathbf{h}} = \int_{\mathbf{h}_x - L}^{\mathbf{h}_x + L} \int_{\mathbf{h}_y - L}^{\mathbf{h}_y + L} \int_{t_0}^{t_f} \rho\,(\mathbf{h}(t))\mathrm{d}x\mathrm{d}y\mathrm{d}t \qquad (2)$$

where t_0 and t_f are the lower and upper bounds of the parametric variable domain, $\mathbf{h}_x$ and $\mathbf{h}_y$ are the x and y components of the ordered triplet generated by $\mathbf{h}(t)$ and $\rho(\mathbf{h}(t))$ the density of the voxel at the location $\mathbf{h}(t)$. For a discrete tomographic volume, this expression may be approximated as:

$$M_{\mathbf{h}} \approx \sum_{i=\mathbf{h}_x - L}^{\mathbf{h}_x + L} \sum_{j=\mathbf{h}_y - L}^{\mathbf{h}_y + L} \sum_{k=t_0}^{t_f} \rho(x_i, y_j, t_k) \cdot L^3 \qquad (3)$$

In order to further attract the space curves to the densest regions of the tomogram, the L^3 term was neglected from equation (3) and the tomographic density was weighted by the inverse square of its distance from the space curve, as:

$$C = \sum_{i=\mathbf{h}_x - L}^{\mathbf{h}_x + L} \sum_{j=\mathbf{h}_y - L}^{\mathbf{h}_y + L} \sum_{k=t_0}^{t_f} \frac{\rho(x_i, y_j, t_k)}{(\mathbf{h}_x - x_i)^2 + (\mathbf{h}_y - y_j)^2} \qquad (4)$$

This cost function C, sometimes combined with a penalty term (see below), was used to optimize the parameters of $\mathbf{h}(t)$ for the initial phase of the algorithm. The optimization was carried out employing two different methods in sequence: i) Particle Swarm Optimization (PSO)[1] and ii) Nelder-Mead simplex method[2]. The former is one of the so-called "global optimization" methods, which performs a wide exploration in the variable space to seek the global optimum of the cost function. Its result depends very little on the starting value and is therefore more suitable for the initial search. However, since it tends to explore with such freedom that it can easily yield extreme and unphysical solutions, its search space was limited in the following way: ω = [-1.5/p, +1.5/p], c_1, c_2 = [-15/p, +15/p], $P_{x^3} = P_{x^2} = P_{z^3} = P_{z^2} = 0$, $P_{x^1} = P_{z^1}$ = [-3, +3], $P_{x^0} = P_{z^0} = \varphi$ = [-∞,

+∞], where p is the voxel length in nm and P_{x^n} and P_{z^n} are polynomial coefficients in x and z, respectively, for the term of order n.

The result from PSO was used as starting point for the second part of the optimization using the simplex method. The latter is more efficient at converging to a nearby local minimum and was thus employed to optimize all the parameters of the space curve. In this phase, a penalty term penalizing high values of ω (> 6/p) (which could generate a single curly path fitting the whole bundle) was introduced to the cost function C. For tomograms like the one in Figure 2, a further term was added, penalizing linear terms in P_x whose absolute value is outside the interval [0.5, 2]. This is to correct for the fact that curves aligned to the y axis can be favored solutions compared to those that run diagonally, because they usually fit a larger portion of the subvolume (apart from the case in which they run along its main diagonal). Therefore, in such densely packed volumes with inevitable experimental noise, it is possible obtain several solutions that run parallel to the y axis and intersect the criss-cross structure, although the latter is clearly prevalent by visual inspection. Alternatively, the y-axis could be rotated align it with one of the criss-cross directions, but this would make it harder to fit the orthogonal direction with the currently employed fitting algorithm.

Once an optimal space curve was found, the tomographic density within 6 nm of the curve was removed from the dataset, a new curve was added and the whole process was repeated, until a designated fraction of the density was removed or a user-defined maximum number of curves were placed.

After placing the adequate number of curves, their associated tomographic density was restored to the analyzed volume and each curve was iteratively refined using the simplex optimizer and adding a repulsion term to the cost function, proportional to the inverse square of the distance:

$$C_s = \sum_{i=\mathbf{h}_x-L}^{\mathbf{h}_x+L} \sum_{j=\mathbf{h}_y-L}^{\mathbf{h}_y+L} \sum_{k=t_0}^{t_f} \frac{\rho(x_i, y_j, t_k)}{(\mathbf{h}_x - x_i)^2 + (\mathbf{h}_y - y_j)^2} - \sum_{\substack{i=1 \\ i \neq s}}^{n} \sum_{j=t_0}^{t_f} k \left[\mathbf{h}_s(t_j) - \mathbf{h}_i(t_j)\right]^{-2} \quad (5)$$

Where s is the curve under consideration (i.e. whose parameters are being optimized), n is the number of fitted space curves and $\mathbf{h}_i(t_j)$ provides the xyz coordinates of the ith space curve at the jth point of an array of t values in [t_0,t_f]. This refinement phase adjusted the curves based on the position of the other curves fitted to the volume. For each iteration, the curves were optimized in

random order to avoid any bias due to the order of the fittings, with the only rule that the first curve of a certain iteration could not be the same as the last curve of the previous iteration. The refinements were carried out until convergence (i.e. the average difference between consecutive iterations is smaller than a threshold) or after a maximum number of iterations had been reached.

Calculation of Nearest-Neighbor distances

For each space curve fitted to a subvolume, the average distance between its position and that of the other curves was approximated by equation (5), and the minimum of these was reported as the nearest-neighbor spacing. For a space curve of the form given by equation (1), the average Euclidian distance average between the ith and j^{th} space curves is approximated over the parametric interval $[t_0,t_f]$ as:

$$\langle D_{i,j}\rangle \approx \frac{1}{n}\sum_{t_0}^{t_f}\sqrt{(\mathbf{h}_i(t)-\mathbf{h}_j(t))^2} \qquad (6)$$

Where $\langle D_{i,j}\rangle$ is the average Euclidian distance, $\mathbf{h}_i(t)$ and $\mathbf{h}_j(t)$ are the *xyz* triples of the ith and j^{th} space curves, respectively, at the parametric value of t, and n is the number of discrete values in the interval $[t_0,t_f]$. Large deviations in the axial direction of the space curve from the parametric axis can result in an overestimate of the spacing calculated by equation (12), thus the nearest-neighbor spacing values represent an approximation of the upper limit of the distance between the curves.

References

1. Kennedy, J., Eberhart, R. Particle Swarm Optimization. *Proc. IEEE- Int. Conf. Neural Networks* **IV**, 1942–1948 (1995).

2. Lagarias, J. C., Reeds, J. A., Wright, M. H., Wright, P. E. Convergence Properties of the Nelder-Mead Simplex Method in Low Dimensions. *SIAM J. Optim.* **9**, 112-147 (1998).
